# Tools for *Mos1*-mediated single copy insertion (mosSCI) with excisable *unc-119(+)* or NeoR (G418) selection cassettes

**DOI:** 10.17912/micropub.biology.000146

**Published:** 2019-08-27

**Authors:** Reta Aram, Kailynn MacGillivray, Chengyin Li, Arneet L Saltzman

**Affiliations:** 1 Department of Cell and Systems Biology, University of Toronto, Toronto, Ontario, Canada

**Figure 1:**
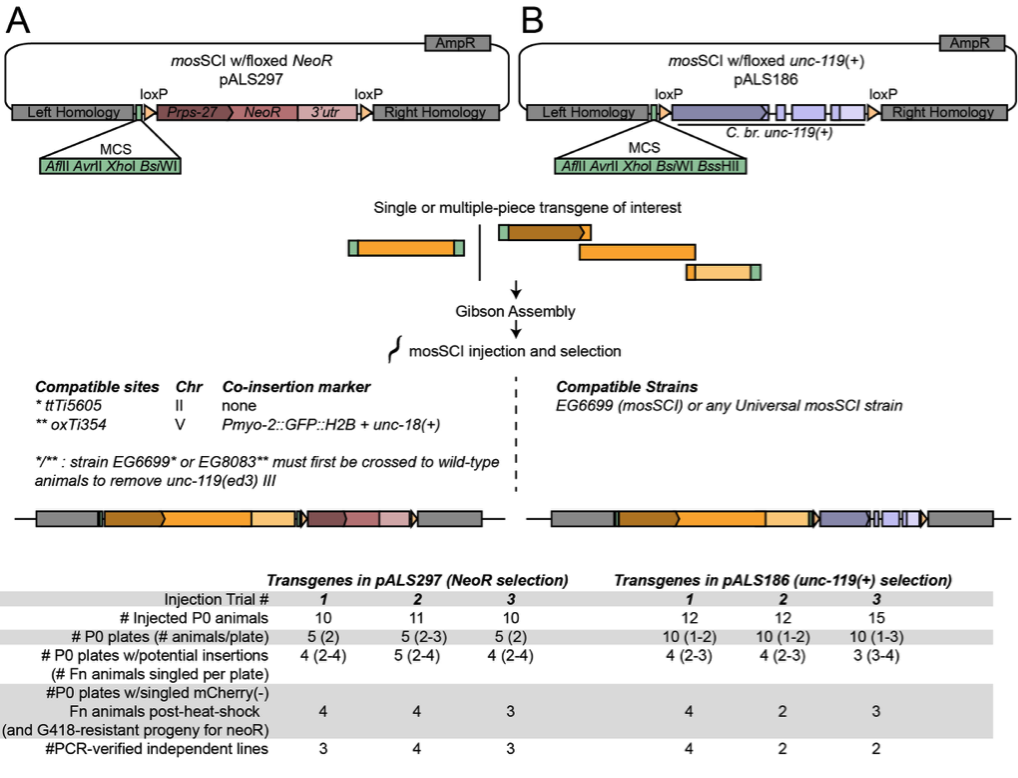
Overview of plasmids for mosSCI at *ttTi5605*-compatible sites using G418 (*NeoR*) (A) or *unc-119*(+) (B) selection cassettes flanked by loxP sites. Both plasmids are modified from pCFJ151 (ttTi5605-MCS) (Frokjaer-Jensen *et al.* 2008). Results of test injections (three different transgene constructs for each plasmid) are shown below the diagrams. The mosSCI “direct injection protocol” was followed, using P*hsp::peel-1* and three *mCherry*(+) co-injection markers to screen against extrachromosomal arrays as described (Frokjaer-Jensen *et al.* 2012)(www.wormbuilder.org). Transgenes in pALS297/*NeoR*or pALS186/*unc-119(+)* were used for mosSCI injections into strains ALS344 or EG6699, respectively (Table 1).

## Description

Insertion of single-copy transgenes is an important tool in *C. elegans* genome engineering. Following the discovery that the *Drosophila* mariner element *Mos1* could mobilize in the *C. elegans* germline (Bessereau *et al.* 2001), tools for efficient *Mos1*-mediated transgene insertion have been developed and widely adopted (Robert and Bessereau 2010). In parallel, the establishment of effective antibiotic selection in *C. elegans* offered increased versatility for genetic crosses and the advantage of injecting into phenotypically wild-type animals (Giordano-Santini *et al.* 2010, Semple *et al.* 2010).

Reliable, site-specific, single-copy transgene insertion remains a key technique for applications such as synthetic reporters and rapid testing of genetic rescue constructs, especially when germline expression is required. The highly developed *Mos1*-mediated single-copy insertion (mosSCI) system is a toolkit of choice for this approach (Frokjaer-Jensen *et al.* 2008, Frokjaer-Jensen *et al.* 2012). The *ttTi5605(mos1)*II insertion site in particular has been used for stable germline expression, and plasmids targeting *ttTi5605* are also compatible with the ‘universal’ mosSCI insertion sites (Frokjaer-Jensen *et al.* 2014).

To add to the suite of tools for targeting *ttTi5605*, we constructed two modified mosSCI plasmids (Figure 1). First, we made a plasmid for *ttTi5605*-mosSCI using G418 selection. Antibiotic selection has been incorporated into the miniMos system for random transgene insertion (Frokjaer-Jensen *et al.* 2014), however existing ttTi5605-mosSCI plasmids use *unc-119(+)* selection. Our plasmid allows use of the *ttTi5605* site without the need for injection into *unc-119(ed3)* mutants, which are more challenging to grow than wild-type animals and do not have optimal germline health on standard *E. coli* OP50 as a food source.

Second, we modified the standard *unc-119* selection-based *ttTi5605*-mosSCI plasmid (pCFJ151) by adding loxP recombination sites flanking the *unc-119(+)* selection cassette (Figure 1B). This change allows excision of the *unc-119(+)* selection cassette using an existing Cre recombinase protocol (Dickinson et al. 2013)*.* Since *unc-119(+)* is a frequently used selection marker, this new feature will add flexibility for construction of strains with multiple transgenes.

For increased versatility, both of the plasmids described here are minimal and do not encode additional tags or fluorescent proteins. Multiple-fragment transgenes can be seamlessly fused and cloned into these plasmids using the Gibson isothermal assembly technique by designing PCR amplicons, synthetic dsDNAs, restriction fragments or oligos with overlapping homologous ends (Gibson *et al.* 2009). We performed three independent test injections using different transgenes and found both of our plasmids to be effective for mosSCI (Figure 1). We also successfully excised the *unc-119(+)* cassette (Figure 1B) using Cre recombinase for two independent insertions (Methods). However, a caveat of the current NeoR plasmid (Figure 1A) is that no additional phenotypic marker was included between the loxP sites. Therefore, unlike *unc-119(+)* excision, which can be easily screened for using a visible phenotype, excision of NeoR would require screening by PCR or by loss of drug resistance. Overall, we hope that these excisable *unc-119(+)* and NeoR mosSCI plasmids will be useful companions to the extensive mosSCI plasmid and strain toolkit (Frokjaer-Jensen *et al.* 2008, Zeiser *et al.* 2011, Frokjaer-Jensen *et al.* 2012, Frokjaer-Jensen *et al.* 2014).

## Reagents

***Plasmid and Strain Construction****.* Plasmid pALS186 (Addgene # 129539; Table 1) was constructed in two steps from pCFJ151 (Addgene # 19330). Briefly, the *C. briggsae unc-119(+)* selection cassette was removed, leaving a modified minimal multiple cloning site (MCS) between the *ttTi5605* insertion homology arms. A PCR amplicon containing *C. br. unc-119(+)* flanked by loxP sites was cloned into the *Bss*HII(*Pte*I)/*Bgl*II sites. To construct plasmid pALS297 (Addgene# 129538; Table 1), the *loxP::C. br. unc-119(+)::loxP* selection cassette was excised from pALS186 by *Bss*HII(*Pte*I)/*Bgl*II digestion and replaced by an *Mlu*I/*Bgl*II-digested PCR amplicon containing the NeoR selection cassette flanked by loxP sites. The NeoR selection cassette (*Prps-27::NeoR::unc-54-3ʹutr*) was amplified from pCFJ910 (Addgene # 44481).

To create strain ALS344, strain EG6699 (CGC; Table 1) was backcrossed 4X to wild-type N2 animals to remove the *unc-119(ed3)* allele. Segregation of *unc-119(ed3)* was followed by the *unc* phenotype. The *ttTi5605(mos1)* insertion site was followed using a three-oligo PCR (o508, o509, o91; Table 1) modified from the Wormbuilder site (http://www.wormbuilder.org/) to simultaneously amplify the ~720 bp product from the *mos1* insertion allele and the 455 bp product from the wild-type allele. PCRs were performed with DreamTaq Green 2X PCR Mastermix (Thermo #K1081) using 1 uL of single worm lysate in a 20 uL reaction at an annealing temperature of 57**°**C.

***Cloning transgenes into mosSCI plasmids****.* Transgenes of interest were assembled from multiple PCR amplicons with ~20 nucleotide overlapping ends into mosSCI plasmids by Gibson Isothermal assembly (Gibson et al. 2009) using a home-made enzyme mix or a kit (NEB #E2621S). For assembly into *Avr*II(*Xma*JI)/*Xho*I-digested pALS297 or pALS186, the first fragment of the transgene is amplified using the forward primer op297-p186AvrIIF (Table 1) and a transgene-specific reverse primer. The last fragment of the transgene is amplified using a transgene-specific forward primer and the reverse primer op297XhoIR or op186XhoIR (Table 1) for assembly into pALS297 or pALS186, respectively.

***Microinjection and screening****.* Worm mosSCI transgenesis was performed according to the direct injection protocol (Frokjaer-Jensen*et al.*, 2012) as described in detail on the Wormbuilder site (http://www.wormbuilder.org/). Transgene-containing mosSCI plasmids were purified by ZymoPURE plasmid miniprep (Zymo #D4209). Co-injected plasmids were purified by HiSpeed Plasmid Midi kit (Qiagen #12643). For *unc-119(+)*selection, plasmids were injected into strain EG6699 (Frokjaer-Jensen *et al*. 2012), which was grown on *E. coli* HB101. For *NeoR*selection, plasmids were injected into strain ALS344 (Table 1) and G418 was added to plates the day after injection as described (Frokjaer-Jensen *et al.* 2014) (125 uL of 25 mg/mL G418 per 35 mm plate).

Following heat-shock and counter-selection for co-injection markers, inserts were verified by PCR using pairs of primers where one primer hybridized to genomic DNA flanking the insertion site and outside of the vector homology arms (o145- 118 nt upstream of left homology arm, or o146- 63 nt downstream of right homology arm) and the second primer hybridized either within the transgene or selection cassette (Table 1). Amplification was carried out using either DreamTaq Green 2X PCR Mastermix (Thermo #K1081; for amplicons <2 kb) or Phusion polymerase (Thermo #F530L; for amplicons >2kb), respectively, according to the manufacturer’s instructions in 20 uL reactions containing 1 uL of a 6uL single worm lysate as the template.

***Excision of the unc-119(+) selection cassette***. The *C. br. unc-119(+)* selection cassette was excised using Cre recombinase as described (Dickinson *et al.*, 2013) using pDD104 (Peft-3::Cre; Addgene # 47551). Briefly, 15-20 animals were injected and moved onto 5-6 plates (3 P0 worms/plate). Approximately 15 F1 animals expressing the mCherry co-injection marker were singled. From the progeny of these animals, *unc* F2 animals were singled to establish excised lines. At least two independent lines were recovered from each of two independent trials. Cre-mediated excision of the NeoR selection cassette has not been tested.

**Table:** 

**Plasmids**
pALS297 (ttTi5605-MCS loxP::*Prps-27::NeoR::unc-54-3ʹutr*::loxP); Addgene # 129538
pALS186 (ttTi5605-MCS loxP::*C.br.unc-119(+)*::loxP); Addgene # 129539

**Strains**
EG6699 *ttTi5605(mos1) II; unc-119(ed3), CGC, note lost extrachromosomal array oxEx1578*
ALS344 *ttTi5605(mos1) II*, this study; EG6699 was crossed 4x to N2 to remove the *unc-119(ed3)* allele

**Primers to follow *ttTi5605(mos1)* II**
o508-mos1F(oJL103) TCTGCGAGTTGTTTTTGCGTTTGAG
o509-ttTi5605F GATTGTTTGACCTGGCGGAACT
o91-ttTi55605R(NM3888) ACGCCCAGGAGAACACGTTAG

**Primers for cloning**
op297-p186AvrIIF TAGAGGGTACCAGAGCTCACCTAGGN*_20-22 _
op297XhoIR GTTATaCGCGCACCGTACGTCTCGAGN*_20-22_
op186XhoIR GTTATgCGCGCACCGTACGTCTCGAGN*_20-22_

**Primers for insert verification**
***5***ʹ***arm, neoR mosSCI***
o145-ttTi5605outerF AGGCAGAATGTGAACAAGACTCGAG (NM3880 + 2nt)
o496-unc54-3ʹutrR GGCCCAGACGTGCGAAGAAATA (or transgene-specific REV primer for long transgenes)
***3***ʹ***arm, unc-119 mosSCI***
o145-ttTi5605outerF AGGCAGAATGTGAACAAGACTCGAG (NM3880 + 2nt)
o147-Cbr-unc-119promR AAGTAGCAGAGCTGGGGAGAAGAA (or transgene-specific REV primer for long transgenes)
***5***ʹ***arm, neoR mosSCI***
o311-NeoRF CTTCTATCGCCTTCTTGACGAG
o146-ttTi5605outerR AATCGGGAGGCGAACCTAACTGT (NM3884 + 2nt)
***3***ʹ***arm, unc-119 mosSCI***
Transgene-specific FOR primer
o146-ttTi5605outerR AATCGGGAGGCGAACCTAACTGT (NM3884 + 2nt)

**Table 1**: List of plasmids, strains and oligonucleotides. Oligonucleotides with NM or JL designations were described by or adapted from Dr. M. Nonet (Washington University School of Medicine in St. Louis) or Dr. J. L. Bessereau (Universitéde Lyon), respectively, based on protocols referenced on the Wormbuilder site (http://www.wormbuilder.org/). *, N_20-22 _ represents 20-22 transgene-specific nucleotides.

## References

[R1] Bessereau JL, Wright A, Williams DC, Schuske K, Davis MW, Jorgensen EM (2001). Mobilization of a Drosophila transposon in the Caenorhabditis elegans germ line.. Nature.

[R2] Dickinson DJ, Ward JD, Reiner DJ, Goldstein B (2013). Engineering the Caenorhabditis elegans genome using Cas9-triggered homologous recombination.. Nat Methods.

[R3] Frøkjær-Jensen C, Davis MW, Ailion M, Jorgensen EM (2012). Improved Mos1-mediated transgenesis in C. elegans.. Nat Methods.

[R4] Frøkjaer-Jensen C, Davis MW, Hopkins CE, Newman BJ, Thummel JM, Olesen SP, Grunnet M, Jorgensen EM (2008). Single-copy insertion of transgenes in Caenorhabditis elegans.. Nat Genet.

[R5] Frøkjær-Jensen C, Davis MW, Sarov M, Taylor J, Flibotte S, LaBella M, Pozniakovsky A, Moerman DG, Jorgensen EM (2014). Random and targeted transgene insertion in Caenorhabditis elegans using a modified Mos1 transposon.. Nat Methods.

[R6] Gibson DG, Young L, Chuang RY, Venter JC, Hutchison CA 3rd, Smith HO (2009). Enzymatic assembly of DNA molecules up to several hundred kilobases.. Nat Methods.

[R7] Giordano-Santini R, Milstein S, Svrzikapa N, Tu D, Johnsen R, Baillie D, Vidal M, Dupuy D (2010). An antibiotic selection marker for nematode transgenesis.. Nat Methods.

[R8] Robert VJ, Bessereau JL (2009). Manipulating the Caenorhabditis elegans genome using mariner transposons.. Genetica.

[R9] Semple JI, Garcia-Verdugo R, Lehner B (2010). Rapid selection of transgenic C. elegans using antibiotic resistance.. Nat Methods.

[R10] Zeiser E, Frøkjær-Jensen C, Jorgensen E, Ahringer J (2011). MosSCI and gateway compatible plasmid toolkit for constitutive and inducible expression of transgenes in the C. elegans germline.. PLoS One.

